# Excellent mechanical properties of taenite in meteoric iron

**DOI:** 10.1038/s41598-021-83792-y

**Published:** 2021-02-26

**Authors:** Shohei Ueki, Yoji Mine, Kazuki Takashima

**Affiliations:** 1 Department of Materials Science and Engineering, Kumamoto University, 2-39-1 Kurokami, Chuo-ku, Kumamoto, 860-8555 Japan; 2 Present Address: Institute of Science and Engineering, Shimane University, 1060 Nishikawatsu, Matsue, Shimane 690-8504 Japan

**Keywords:** Characterization and analytical techniques, Mechanical properties

## Abstract

Meteoric iron is the metal that humans first obtained and used in the earliest stage of metal culture. Advances in metallographic analysis techniques have revealed that meteoric iron largely comprises kamacite, taenite, and cohenite, which correspond to ferrite, austenite, and cementite in artificial steel, respectively. Although the mechanical properties of meteoric irons were measured previously to understand their origin and history, the genuine mechanical properties of meteoric iron remain unknown because of its complex microstructure and the pre-existing cracks in cohenite. Using micro-tensile tests to analyse the single-crystalline constituents of the Canyon Diablo meteorite, herein, we show that the taenite matrix exhibits excellent balance between yield strength and ductility superior to that of the kamacite matrix. We found that taenite is rich in nitrogen despite containing a large amount of nickel, which decreases the nitrogen solubility, suggesting that solid-solution strengthening via nitrogen is highly effective for the Fe–Ni system. Our findings not only provide insights for developing advanced high-strength steel but also help understand the mysterious relationship between nitrogen and nickel contents in steel. Like ancient peoples believed that meteoric iron was a gift from the heavens, the findings herein imply that this thought continues even now.

## Introduction

Meteoric irons, known as Fe–Ni alloys with various nickel contents from a minimum of 5 up to 60 mass%, can be classified into several types exhibiting different microstructural features based on their overall nickel content^1^. The main phases of meteoric iron are kamacite with a body-centred cubic (BCC) structure, taenite with a face-centred cubic (FCC) structure, and cohenite with an orthorhombic structure, which correspond to α-ferrite, γ-austenite, and Fe_3_C cementite in artificial steel, respectively^2–6^. The unique microstructure of meteoric iron is formed by the nucleation and growth of kamacite from taenite during the slow cooling of the parent body^1^, with the cooling period for a 1 K temperature decrease estimated as a few hundred to thousands of years^1^. Despite the huge difference in cooling rates between artificial steel and meteoric iron (more than 13 orders of magnitudes), the crystallographic features of the FCC to BCC transformation mechanism are similar in artificial steel and meteoric iron^2–5^. However, there have been few studies on the mechanical properties of meteoric iron^7–12^. Most measurements of their mechanical properties determined tensile strengths less than ca. 100 MPa, which are relatively low values compared to that of artificial iron-nickel alloys^8,11^. This is because pre-existing cracks and cavities in the body of meteoric iron hinder the measuring of the strength and ductility of their constituents. To measure the mechanical properties of metallic microconstituents, micropillar-compression tests are frequently perfomed^13–15^. This method is suitable for examining the dislocation motion and sample size effect on the deformation behaviour. However, the deformation restraint on the bottom of the pillar prevents the complete uniaxial stress loading throughout the specimen, making it difficult for the accurate measurement of the yield strength, strain hardening, and elongation. In recent times, advances in micro-mechanical testing techniques have reduced tensile-test specimens to the microstructural scale, i.e. several tens of micrometres, which has allowed for the successful characterisation of steel tensile properties and deformation behaviours^16–20^. Therefore, we applied the micro-tensile testing technique to the mechanical characterisation of genuine stress–strain behaviours in the constituents of a meteoric iron.

## Results and discussion

### Microstructural characterisation

**Figure 1:**
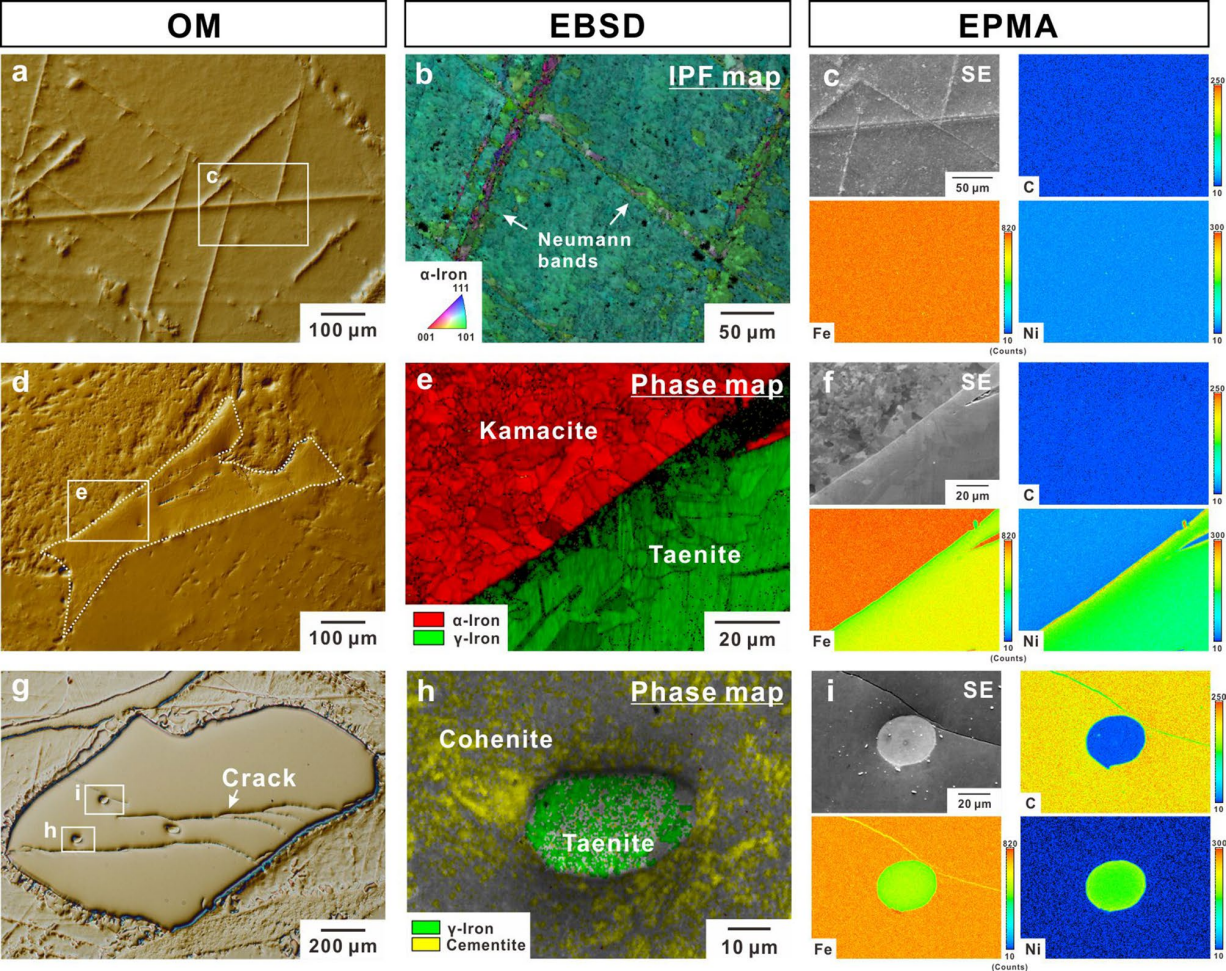
Microstructural characterisation of Canyon Diablo meteorite. (**a**,**d**,**g**) Optical micrographs showing kamacite, taenite, and cohenite, respectively. (**b**,**e**,**h**) EBSD maps in kamcite, taenite/kamacite boundary, and cohenite, respectively. (**c**,**f**,**i**) Comparison of secondary electron image and corresponding elemental maps of carbon, iron, and nickel in the areas shown in (**a**), (**e**), and (**g**), respectively. Colour bars in elemental maps indicate the characteristic X-ray intensity proportional to the elemental concentration.

Figure 1 shows the optical micrographs, electron back-scatter diffraction (EBSD) patterns, and electron probe microanalysis (EPMA) maps of the typical constituents in a Canyon Diablo meteorite sample. Figure 1a shows the typical microstructural morphology which accounts for the majority of this meteoric iron, and Fig. 1b shows the areas determined as α-iron via EBSD analysis. Additionally, Neumann bands were observed, which are deformation twins in the BCC crystal, as previously reported for some meteoric irons^2,3^. According to the compositional mapping of alloying elements by EPMA shown in Fig. 1c, there was no segregation of alloying elements in the boxed area in Fig. 1a. Quantitative analysis of the alloying elements (Extended Data Table 1) in the area shown in Fig. 1c revealed that the α-phase could be regarded as ferritic iron containing approximately 6 at.% Ni, i.e. kamacite. A small fraction of taenite (determined by γ-iron in the EBSD analysis) was observed as shown in Fig. 1d,e. The compositional mapping of alloying elements in taenite revealed that the iron content decreased and nickel content increased upon approaching the taenite/kamacite boundary (Fig. 1f). Beyond the area near the taenite/kamacite boundary, taenite was composed of 1.61C, 5.18 N, 0.03P, 72.60Fe, 0.23Co, and 20.35Ni in at.% (Extended Data Table 1). Additionally, cohenite (determined by cementite in the EBSD analysis) was frequently observed, as shown in Fig. 1g,h. Cohenite was composed of 20.66C, 0.53 N, 0.001P, 77.48Fe, 0.17Co, and 1.16Ni in at.%, corresponding to Fe_3_C cementite (Extended Data Table 1). Furthermore, numerous cracks and small areas of taenite were observed in cohenite (Fig. 1g–i). A micro-tensile test of single-crystalline cohenite revealed that the cleavage fracture occurred on the (011) plane without plastic deformation at the maximum tensile strength of 2.4 GPa (Extended Data Fig. 1). We expect that these are why the previous measurements of the tensile properties of meteoric iron^8,11^ exhibited low strength levels, because the brittle materials are sensitive to defects. Subsequently, based on the microstructural determination by EBSD and EPMA analyses, we performed micro-tensile tests for kamacite and taenite of the Canyon Diablo meteorite sample.

### Stress–strain curves

**Figure 2:**
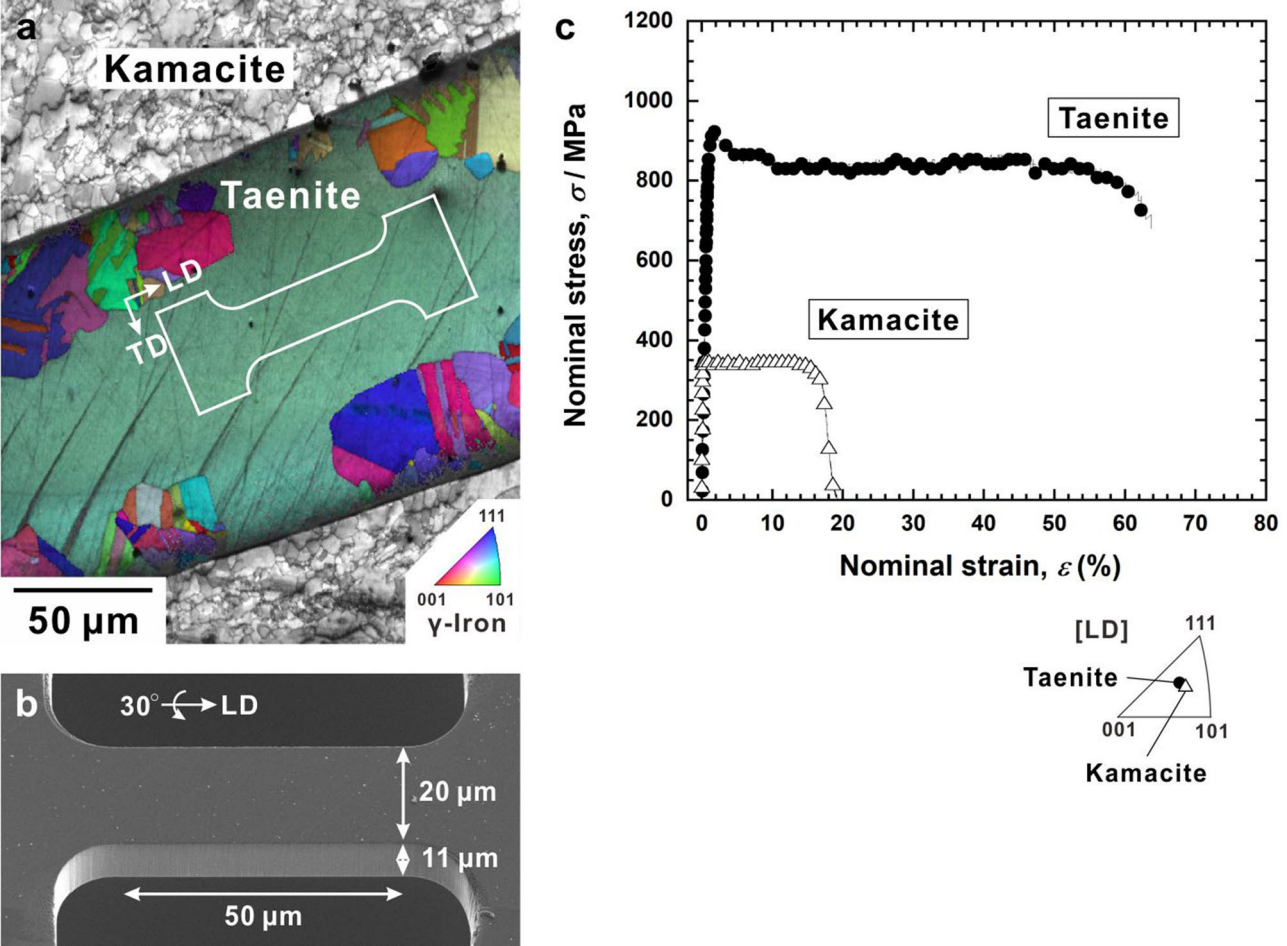
Preparation of micro-tensile specimen and obtained stress–strain behaviours. (**a**) Inverse pole figure map overlaid by schematic illustration of micro-tensile specimen in taenite. LD and TD denote the loading and transverse directions, respectively. (**b**) Scanning electron microscopy image showing the shape and dimensions of the micro-tensile specimen. (**c**) Nominal stress–strain, *σ*–*ε*, curves for the taenite and kamacite specimens. The stereographic triangle shows the LDs in the taenite and kamacite specimens.

Figure 2a,b show an example of preparing a micro-sized tensile specimen for taenite. As illustrated in the inverse pole figure map of taenite (Fig. 2a), a micro-tensile specimen with a gauge section of 11 × 20 × 50 μm^3^ was fabricated using a focused ion beam (Fig. 2b) so that the gauge part was completely single crystalline. We chose this length scale because no significant size effect will appear down to this specimen size^21–23^. Thus, we can evaluate the mechanical properties of the microstructural constituents, which are equivalent to those of the bulk specimen. A single-crystalline specimen of kamacite was prepared in the same way. Figure 2c shows the stress–strain curves obtained by micro-tensile testing of the single-crystalline taenite and kamacite specimens with their loading directions (LDs) nearly parallel to the [123] direction. For kamacite, the yield strength and elongation-to-failure were 350 MPa and 19%, respectively, which are in good agreement with the previous estimated yield strength of 335 MPa and elongation-to-failure of 19%, obtained by the tensile testing of a Gibeon meteorite^7^ with a Widmanstätten structure and coarse kamacite widths. Meanwhile, the yield strength and elongation-to-failure of taenite were 935 MPa and 65%, respectively. Although there have been no reports on high-nitrogen alloying in nickel-rich austenitic steels because nickel decreases the nitrogen solubility^24^, introducing high levels of nitrogen into Fe–Cr–Mn alloys^25,26^ is well known as a remarkable strengthening method via solid-solution strengthening. For example, the yield strength of the Fe–24Cr–10Mn–1.43 N alloy (mass%), which contains an amount of nitrogen comparable to that in the taenite in this study (1.35 N mass%), was measured as 830 MPa^26^. Therefore, the high yield strength of taenite was presumably attributable to a solid-solution-strengthening mechanism via the interaction between dislocations and interstitial nitrogen atoms^27^.

### Micro-tensile behaviours

**Figure 3:**
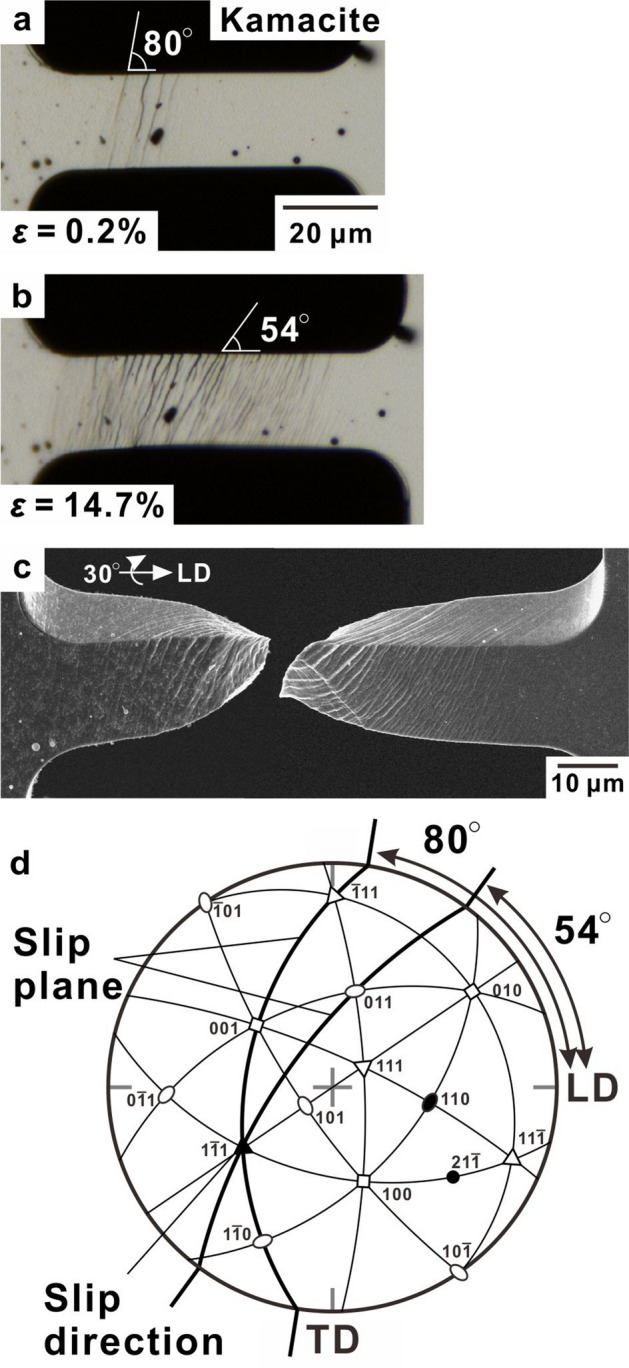
Deformation and fracture behaviour in the single-crystalline kamacite specimen. (**a**,**b**), Optical micrographs captured during deformation at nominal strains, *ε*, of 0.2% and 14.7%, respectively. (**c**) Scanning electron microscopy image showing the fracture morphology. (**d**) Stereographic projection of the kamacite specimen in the initial state with related slip systems.

**Figure 4:**
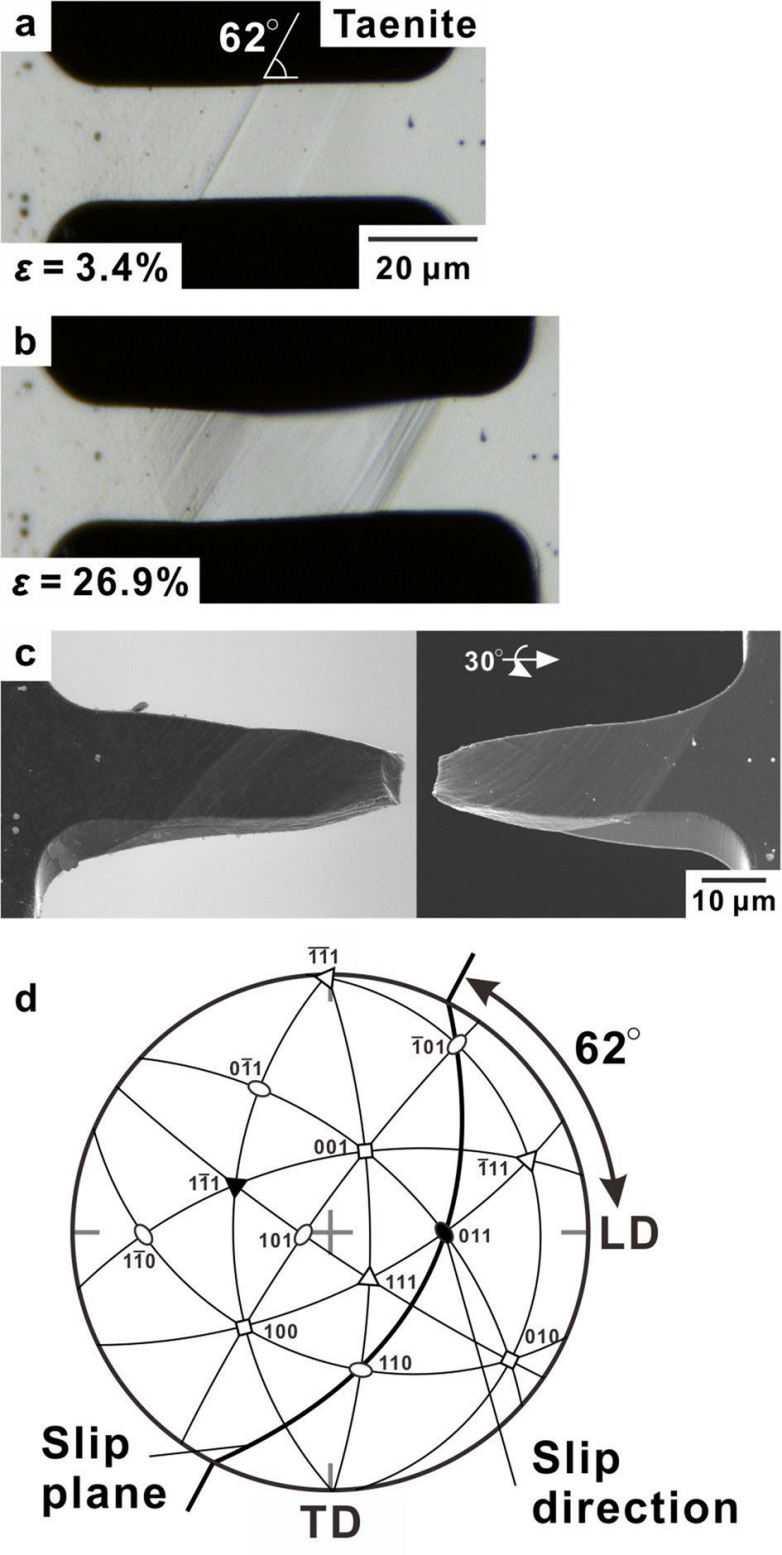
Deformation and fracture behaviour in the single-crystalline taenite specimen. (**a**,**b**) Optical micrographs captured during deformation at nominal strains, *ε*, of 3.4% and 26.9%, respectively. (**c**) Scanning electron microscopy image showing the fracture morphology. (**d**) Stereographic projection of the taenite specimen in the initial state with related slip systems.

Figure 3 shows the deformation and fracture morphology of the kamacite micro-tensile specimen. Slip steps formed at inclination angles of 80° and 54° with respect to the LD after the onset of yielding (Fig. 3a,b), and they propagated while maintaining a constant stress level (Supplementary Video 1). Finally, chisel-edge-type fracture with significant necking occurred (Fig. 3c). Figure 3d shows a stereographic projection of the kamacite specimen based on the EBSD analysis, which indicates that the observed slip steps correspond to the primary slip systems (110) [1$\overline{1}$1] and (21$\overline{1}$) [1$\overline{1}$1], with Schmid factors of 0.47 and 0.49, respectively. This suggested that cross slips occurred on the favourable slip planes with the same slip direction and that the kamacite matrix deformed based on Schmid’s law. Figure 4 shows the deformation and fracture morphology of the taenite micro-tensile specimen. In the taenite specimen, a linear slip step formed at an inclination angle of 62° with respect to the LD at the onset of yielding (Fig. 4a), and thereafter the deformation band extended throughout the gauge part of the specimen (Fig. 4b), like the Lüders deformation (Supplementary Video 2). Finally, the specimen fractured with significant necking (Fig. 4c). The linear slip step observed in the taenite specimen corresponds to the primary slip system of (1$\overline{1}$1) [011], with a Schmid factor of 0.47 (Fig. 4a,d). It should be noted that the observed slip steps in the kamacite and taenite specimens exhibited wavy and linear morphologies, respectively. Active slip systems in BCC crystals are in the <111> direction on the {110}, {112}, and {123} planes, whereas those in FCC crystals are in the <110> direction on the {111} planes. Put simply, kamacite (BCC) has more active slip systems than taenite (FCC). Therefore, the difference in slip behaviour was attributable to the difference in the number of slip systems in kamacite and taenite.

**Figure 5:**
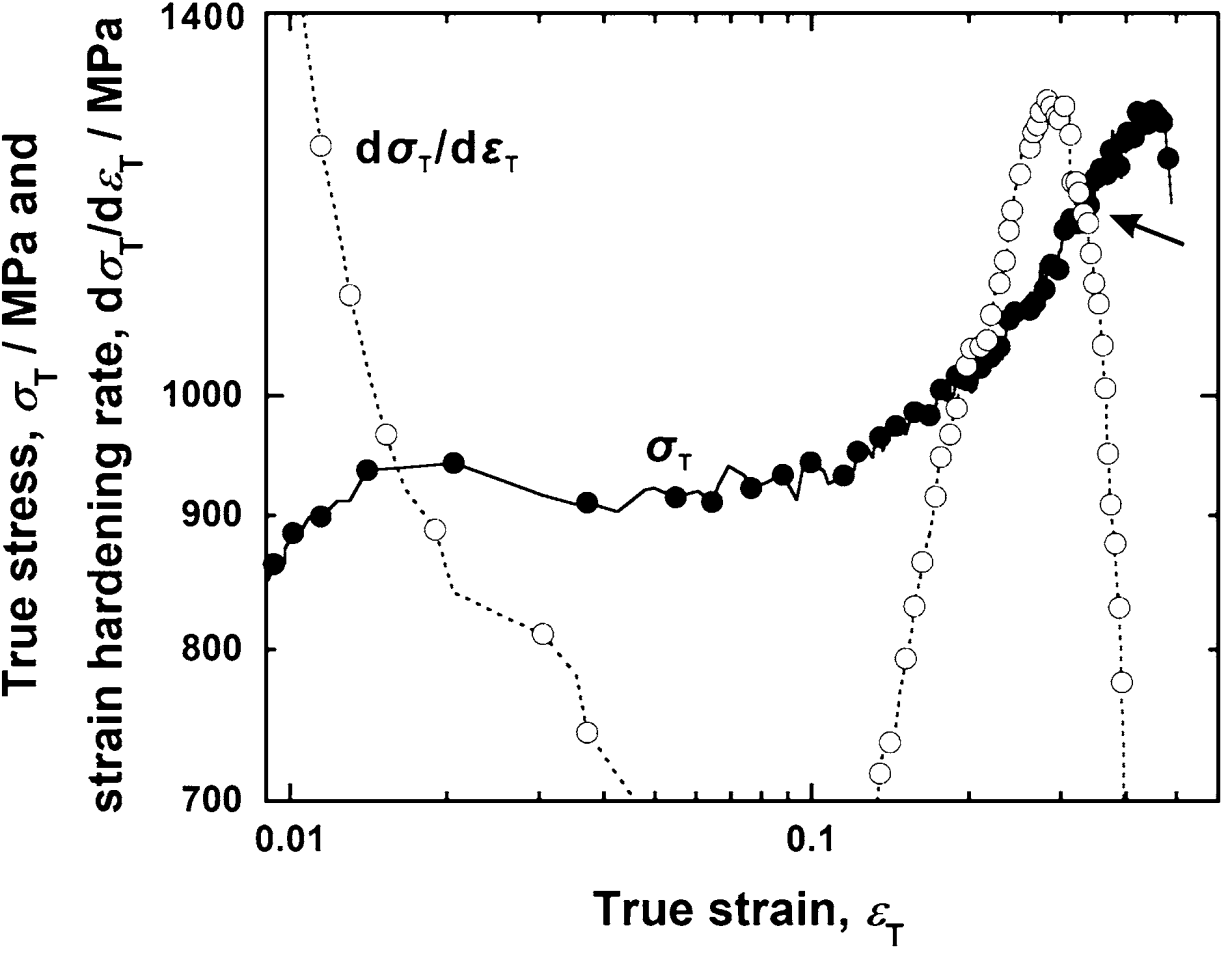
Relationship between true stress and strain hardening rate plotted against true strain for the taenite specimen. *σ*_T_ and *ε*_T_ were calculated using the following equations: *σ*_T_ = *σ* (1 + *ε*) and *ε*_T_ = ln (1 + *ε*). Since these equations can be applied in the case of isotropic shrinkage due to uniaxial deformation under the constant-volume deformation assumption, the calculations are invalid during local deformation regime and beyond the plastic instability.

The stress–strain curve of taenite (Fig. 2c) revealed a high yield strength without a loss in the good ductility. Figure 5 shows the relationships between the true stress, *σ*_T_, and strain hardening rate, d*σ*_T_/d*ε*_T_, plotted against the true strain, *ε*_T_, for the taenite specimen. Generally, single-crystalline FCC metals exhibit a transition from the easy-glide stage to linear-hardening stage owing to the interaction of multiple slip systems. In the taenite specimen, indeed, single slip gliding proceeds throughout the gauge part of the specimen (Fig. 4a,b), followed by the strain hardening concurrent with the activation of the secondary slip system (Fig. 5). The onset of plastic instability can be determined by the Considѐre’s condition: d*σ*_T_/d*ε*_T_ ≤ *σ*_T_. In the taenite specimen having high yield strength, the strain hardening stage is short because the stress level exceeds the strain hardening rate at an early stage of strain hardening (indicated by the arrow in Fig. 5). In austenitic stainless steels, nitrogen increases not only the strength but also the strain hardening rate^25^. This indicates that the occurrence of local necking is suppressed in the easy-glide stage, which enables uniform deformation up to a high-strain region.

Micro-tensile tests of the Canyon Diablo meteorite specimens revealed that the nickel-rich austenite with an ultra-high nitrogen content exhibited an excellent relationship between yield strength and ductility. Generally, advanced high-strength austenitic steels, which combine a high strength and moderate ductility via transformation-induced plasticity and twin-induced plasticity, exhibit tensile strengths exceeding ca. 800 MPa. It is a major challenge to increase the yield strength of advanced high-strength austenitic steels because of a risk of hydrogen embrittlement under severe external conditions^28^. Therefore, we anticipate that our findings will aid in the development of advanced high-strength steels, and motivate research into understanding the relationship between nitrogen and nickel contents in steel.

## Methods

### Material and microstructural characterisation

The material used in this study was a Canyon Diablo meteorite (type IAB, coarse octahedrite). Small-cut samples were polished with emery paper and a colloidal SiO_2_ paste. Compositional mappings in the constituents of the meteorite were conducted using a JEOL (JCM-5700) scanning electron microscope (SEM) equipped with an electron probe micro-analyser (EPMA, Shimadzu 1720), and operated at a beam current of 0.1 μA and accelerating voltage of 15 kV. All the measurements were obtained using the Kα signal of C, N, P, Fe, Co, and Ni. Quantitative 12-point analyses were performed in each characteristic phase using a focused beam at a beam current of 0.05 μA and accelerating voltage of 15 kV. The ZAF correction method was applied for the quantitative analysis of chemical compositions using reference samples of Cr_3_C_2_, AlN, GaP, Fe, Co, and Ni for each element (C, N, P, Fe, Co, and Ni, respectively). The methods of Duncumb-Reed, Philibert, and Reed were used for correcting the effects of atomic number (Z), absorption (A), and fluorescence (F), respectively. The average values were calculated from the results of the 12-point analyses for each element in phases to estimate the chemical compositions. After the EPMA analysis, the crystal orientation was determined at a scanning step size of 0.4 μm using an SEM instrument equipped with an EBSD detector and orientation imaging microscopy software (TSL OIM v.7.1.0). A clean-up procedure was applied to all the EBSD images to adjust single points having misorientations greater than 5° in comparison with their neighbours. Additionally, points with a confidence index lower than 0.1 were excluded from the analysis based on Field’s study^29^.

### Micro-tensile tests

The samples were thinned to a thickness of approximately 20 μm using emery paper, and both the surfaces were then mirror-finished with a colloidal SiO_2_ paste. Micro-tensile specimens with a gauge section of 11 × 20 × 50 μm^3^, 18 × 20 × 50 μm^3^, and 22 × 20 × 50 μm^3^ were fabricated using a focused ion beam for taenite, kamacite, and cohenite, respectively. In the regime of this length scale, no difference was observed in the strength^22,23^ due to the sample size effect which occurs in micropillar compression^21^. Single-crystalline specimens were prepared with their LDs approximately parallel to the [123] direction for kamacite and taenite, and to the [011] direction for cohenite. Tensile tests were performed at room temperature under laboratory atmospheric conditions with a displacement rate of 0.1 μm s^–1^, corresponding to a strain rate of 2 × 10^–3^ s^–1^. The set-up has been described in more detail in a previous study^30^. The gauge section of the tensile specimen was monitored during tensile testing using an optical microscope to dynamically measure the strain as a function of time.

## Supplementary information

Supplementary Information.

Supplementary Video 1.

Supplementary Video 2.

## Data Availability

All data generated or analysed during this study are included in the published article and Supplementary Information and are available from the corresponding author upon reasonable request.
