# Supplementary material for: Excellent mechanical properties of taenite in meteoric iron
Source: Sci Rep. 2021 Feb 26;11:4750. doi: 10.1038/s41598-021-83792-y (PMC7910554; doi:10.1038/s41598-021-83792-y)
Supplement: Supplementary file 1 — Supplementary Information. [file 41598_2021_83792_MOESM1_ESM.doc]

**Excellent mechanical properties of taenite in meteoric iron**

Shohei Ueki^†^, Yoji Mine*, Kazuki Takashima

Department of Materials Science and Engineering, Kumamoto University, 2-39-1 Kurokami, Chuo-ku, Kumamoto 860-8555, Japan

^†^ Present address: Institute of Science and Engineering, Shimane University, Japan

* Correspondence to: [mine@msre.kumamoto-u.ac.jp](mailto:mine@msre.kumamoto-u.ac.jp)

**This supplementary information includes:**

Supplementary Text

Reference

Extended Data Fig. 1

Extended Data Table 1

Captions for Supplementary videos 1 and 2

**Supplementary Text**

Micro-tensile test on the single-crystalline Cohenite

Extended Data Fig. 1 shows the stress–strain curve obtained by micro-tensile testing and fracture surface of single-crystalline cohenite specimen with the loading direction (LD) nearly parallel to [011] direction. The specimen fractured at the maximum tensile strength of 2385 MPa without plastic deformation. Fracture surface was almost perpendicular to the LD and exhibited typical cleavage feature with river pattern as shown in Extended Data Fig. 1. These findings indicate that the cleavage fracture parallel to (011) plane occurred in the single-crystalline cohenite specimen, which is in good agreement with the previous report^31^ of Vickers indentation study of cementite in Fe–5.8Cr–4.8C (in mass%). We expect that application of micro-tensile testing technique to the mechanical characterization of cohenite remarkably advances the research clarifying the deformation and fracture mechanisms of cementite.

**Reference**

31. Inoue, A., Ogura, T. and Matsumoto, T. Deformation and fracture behaviours of cementite. *Trans. JIM* **17**, 663–672 (1976).

**Extended Data** **Fig. 1 | Stress–strain and fracture behaviours in the single-crystalline cohenite specimen. a**, Nominal stress–strain, *σ*–*ε*, curve for the single-crystalline cohenite specimen with the loading direction nearly parallel to [011] direction. **b**
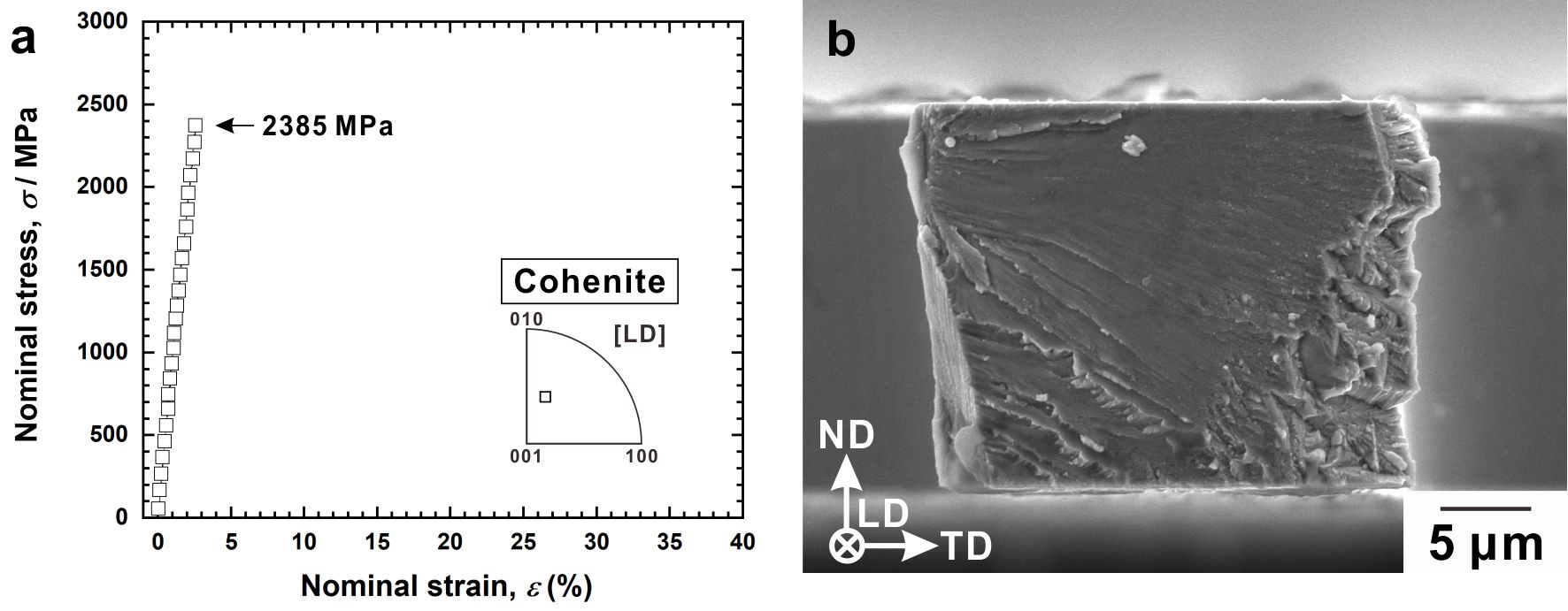
, Scanning electron microscopy image showing the (011) cleavage fracture surface of cohenite specimen.

**Extended Data Table 1 | Chemical compositions of kamacite, taenite, and cohenite in Canyon Diablo meteorite**

| (at.%) | | | | | | |
| --- | --- | --- | --- | --- | --- | --- |
|  | C | N | P | Fe | Co | Ni |
| Kamacite | 1.10 | 0.77 | 0.19 | 90.88 | 0.65 | 6.41 |
| Taenite | 1.61 | 5.18 | 0.03 | 72.60 | 0.23 | 20.35 |
| Cohenite | 20.66 | 0.53 | 0.001 | 77.48 | 0.17 | 1.16 |

**Supplementary Video 1 | Micro-tensile behaviour of the kamacite specimen in Canyon Diablo meteorite.** In-situ optical micrography showing the yielding, plastic deformation, and fracture with the corresponding nominal stress–strain curve in the single-crystalline kamacite specimen with the [123] loading direction.

**Supplementary Video 2 | Miro-tensile behaviour of the taenite specimen in Canyon Diablo meteorite.** In-situ optical micrography showing the yielding, plastic deformation, and fracture with the corresponding nominal stress–strain curve in the single-crystalline taenite specimen with the [123] loading direction.
